# A novel mitochondrial protein is required for cell wall integrity, auxin accumulation and root elongation in Arabidopsis under salt stress

**DOI:** 10.1007/s44154-022-00036-3

**Published:** 2022-02-08

**Authors:** Zheping Yu, Yuying Ren, Jianwei Liu, Jian-Kang Zhu, Chunzhao Zhao

**Affiliations:** 1 Institute of Horticulture, Zhejiang Academy of Agricultural Sciences, Hangzhou, 310021 China; 2 Shanghai Center for Plant Stress Biology, CAS Center for Excellence in Molecular Plant Sciences, Chinese Academy of Sciences, Shanghai, 200032 China

**Keywords:** Salt stress, Auxin, Reactive oxygen species, Plant cell wall, Arabidopsis

## Abstract

Maintenance of root elongation is beneficial for the growth and survival of plants under salt stress, but currently the cellular components involved in the regulation of root growth under high salinity are not fully understood. In this study, we identified an Arabidopsis mutant, *rres1*, which exhibited reduced root elongation under treatment of a variety of salts, including NaCl, NaNO_3_, KCl, and KNO_3_. *RRES1* encodes a novel mitochondrial protein and its molecular function is still unknown. Under salt stress, the root meristem length was shorter in the *rres1* mutant compared to the wild type, which was correlated with a reduced auxin accumulation in the mutant. Reactive oxygen species (ROS), as important signals that regulate root elongation, were accumulated to higher levels in the *rres1* mutant than the wild type after salt treatment. Measurement of monosaccharides in the cell wall showed that arabinose and xylose contents were decreased in the *rres1* mutant under salt stress, and application of boric acid, which is required for the crosslinking of pectic polysaccharide rhamnogalacturonan-II (RG-II), largely rescued the root growth arrest of the *rres1* mutant, suggesting that RRES1 participates in the maintenance of cell wall integrity under salt stress. GUS staining assay indicated that the *RRES1* gene was expressed in leaves and weakly in root tip under normal conditions, but its expression was dramatically increased in leaves and roots after salt treatment. Together, our study reveals a novel mitochondrial protein that regulates root elongation under salt stress via the modulation of cell wall integrity, auxin accumulation, and ROS homeostasis.

**Supplementary Information:**

The online version contains supplementary material available at 10.1007/s44154-022-00036-3.

## Introduction

Salinity is one of the abiotic stresses that adversely affect plant growth and agricultural productivity worldwide. During the last three decades, the molecular mechanisms of salt stress response in plants have been extensively studied (Gong et al., 2020). The detrimental effects of salt stress on plant growth and development can be divided into three aspects: osmotic stress, ion toxicity, and oxidative stress (Hasegawa et al., 2000; Roy et al., 2014; Hazman et al., 2015). After being exposed to high salinity, rapid elevation of ions surrounding root cells immediately triggers osmotic stress (Roy et al., 2014). Osmotic stress affects many developmental and physiological processes, including cell volume and turgor pressure, cell dehydration, cell division and expansion, and photosynthesis rate (Gong et al., 2020). The second phase is ionic stress and it occurs when ions are transported into plant cells. High concentration of Na^+^ in the cytoplasm is toxic to enzymes and thus causes cell growth arrest and even cell death (Hasegawa et al., 2000; Gong et al., 2020). To cope with the toxic effect, excessive ions need to be extruded from the cytoplasm to the outside of cells or compartmentalized into the vacuole. Meanwhile, maintenance of Na^+^ and K^+^ homeostasis is critical for attenuating the adverse effect of Na^+^ (Zhu, 2003). High salinity also leads to the production of excessive reactive oxygen species (ROS), which triggers oxidative stress in plant cells (Zhu et al., 2007; Hazman et al., 2015). Appropriate concentration of ROS can be used as important signaling molecules in response to salt stress (Yang et al., 2014; Yang & Guo, 2018). However, excessive accumulation of ROS causes oxidative stress, leading to damages of DNA, proteins, and lipids (Moldovan & Moldovan, 2004; Genisel et al., 2014; Golldack et al., 2014).

Root is essential for plants to uptake water and nutrients to support the growth and development of aerial organs. Under salinity conditions, root is the first organ that suffers stress. High salinity severely inhibits root growth in a vast majority of plant species, including cotton (Zhong and LÄUchli, 1993), rice (Lin & Kao, 1996), Arabidopsis (Wu et al., 1996), maize (Rodriguez et al., 1997)*,* soybean (Phang et al., 2008), wheat (Rahnama et al., 2011), and barley (Shelden & Roessner, 2013). High salinity affects primary root growth by inhibiting cell division and cell expansion (Gong et al., 2020), and auxin signaling pathway is shown to be involved in the regulation of these processes (Tomas et al., 2020). Under salt stress, auxin level and the expression of auxin transporters are reduced, which results in reduced root meristem activity and primary root growth inhibition (Liu et al., 2015). Therefore, understanding of the regulatory mechanisms of auxin signaling under salt stress will be useful for future biotechnological application to increase salt tolerance in plants (Tomas et al., 2020).

In plants, cell wall not only determines cell expansion and growth but also provides a mechanical protection against abiotic stress (Wolf et al., 2012). Accumulating evidence indicates that maintenance of cell wall integrity is critical for salt tolerance in plants. Many mutants that are disrupted in cell wall biosynthesis, modification, or perception exhibit an increased sensitivity to salt stress. Mutation of *SOS5* gene, which encodes an apoplastic ﻿arabinogalactan protein (AGP)-like protein, results in a severe root growth arrest under salt stress (Shi et al., 2003). *CESA6* encodes one of the cellulose synthases that are essential for cellulose biosynthesis. Due to abnormal cellulose biosynthesis, the *cesa6* mutant displays severe root growth inhibition and cell swelling under salt stress. In agreement with this finding, it was reported that cellulose synthase-like protein SOS6 and companion of cellulose synthase 1 and 2 (CC1 and CC2) are required for root and hypocotyl elongation under salt stress (Zhu et al., 2010; Endler et al., 2015). Our recent study reported that *MUR4*, which is required for UDP-Ara biosynthesis, is involved in the maintenance of cell wall integrity under salt stress. The *MUR4* gene mutation leads to abnormal cell-cell adhesion and reduced root elongation under salt stress (Zhao et al., 2019). To avoid the disruption of cell wall integrity during cell expansion and in response to environmental stresses, plants have developed various sensors to continuously monitor cell wall status. FERONIA (FER), a plasma-membrane localized receptor kinase, is considered as one of the cell wall integrity sensors. Mutation of *FER* gene results in enhanced leaf bleaching and root cell swelling under salt stress, and it was proposed that FER participates in the regulation of cell wall repair under salt stress via a Ca^2+^-mediated signaling pathway (Deslauriers & Larsen, 2010; Feng et al., 2018). In this study, through a high-throughput genetic screen of *Arabidopsis* mutants that are hypersensitive to salt stress, we identified a mutant that displayed slower root elongation under salt stress compared with the wild type plants. Analysis of monosaccharides showed that arabinose and xylose contents in the cell wall were reduced in the mutant after salt treatment, and application of exogenous boric acid largely rescued the root growth inhibition of the mutant, supporting the importance of cell wall integrity in salt tolerance. Moreover, ROS and auxin homeostasis were also disrupted in the mutant under salt stress.

## Results

### RRES1 is required for the regulation of root elongation under salt stress

To identify genes that participate in the regulation of salt tolerance, we performed a genetic screen for *Arabidopsis* mutants that were generated by EMS-mediated mutagenesis (Fujii et al., 2011; Wu et al., 1996). Multiple mutants that showed reduced root elongation under high salinity were identified, and here we report one of these mutants, named *19–1*. The *19–1* mutant showed slightly reduced root elongation when grown on MS medium, but exhibited strong root growth inhibition when grown on MS medium supplemented with 100 mM NaCl (Fig. S1a and b). To identify mutated gene in the *19–1* that was responsible for salt-hypersensitivity, we backcrossed *19–1* with its parent plant and performed bulk segregant analysis (BSA) using F_2_ population. Mapping result revealed a significant peak at the end of the second chromosome (Fig. S1c). By searching for all the mutations in this region, we found a C199T mutation in *AT2G45320* gene, which led to a pre-mature stop at Gln67. *AT2G45320* is a novel gene that has not been reported before. As this gene mutation led to reduced root elongation under salt stress, we designed it as *RRES1* (*Reduced* *Root* *Elongation under* *Salt Stress 1*). Alignment of the protein sequence of RRES1 against the Arabidopsis database showed that the Arabidopsis contains only one copy of RRES1. According to the Arabidopsis Information Resource (TAIR, http://arabidopsis.org) annotation, *RRES1* is predicted to encode a polyphosphatidylinositol phosphatase. However, alignment of this protein against the protein database in the NCBI, the orthologs of RRES1 are annotated as nucleotide-diphospho-sugar transferases.

**Fig. 1:**
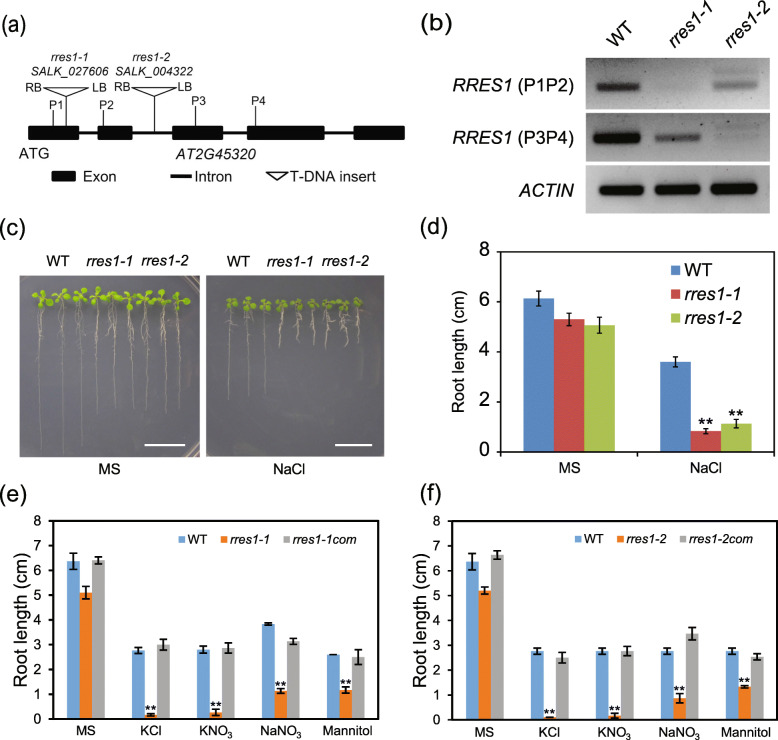
RRES1 is required for the regulation of root elongation under salt stress. **a** Diagram of T-DNA insertion sites in two independent *rres1* mutant alleles. Triangles represent the T-DNA insertion sites in *RRES1* gene. Black rectangles represent exon and black lines represent intron. **b** Transcript level of *RRES1* in wild-type, *rres1–1*, and *rres1–2* was evaluated by RT-PCR. *ACTIN* gene was used as an internal control. **c** Phenotype of the wild type, *rres1–1* and *rres1–2* grown on Murashige and Skoog (MS) media supplemented with or without 100 mM NaCl. Scale bar = 2.5 cm. **d** Quantification of root length of the wild type and *rres1* mutants. Five-day-old seedlings were transferred to MS or MS + 100 mM NaCl media and grown for 7 days, and the length of newly developed roots was measured. Values indicate means ± SD (*n* = 5). Asterisks indicate statistically significant differences (**p < 0.01 by Student’s *t* test). **e-f** Quantification of root length of the wild type, *rres1* mutant and complementation lines after being transferred to MS media supplemented with 100 mM KCl, 80 mM KNO_3_, 80 mM NaNO_3_, or 0.5 M mannitol for 7 days. Asterisks indicate statistically significant differences (**p < 0.01 by Student’s *t* test)

To verify that *RRES1* is the gene that is required for root growth under salt stress, we ordered two independent T-DNA insertion lines SALK_027606 and SALK_004322, named as *rres1–1* and *rres1–2*, from the Arabidopsis Stock Center (ABRC). In these two lines, the T-DNAs were inserted in the first exon and second intron of the *RRES1* gene, respectively (Fig. 1a). RT-PCR assay showed that the *RRES1* gene expression was largely reduced in both the *rres1–1* and *rres1–2* mutant alleles (Fig. 1b). Both the *rres1* mutants showed similar developmental phenotypes as the wild type, including normal rosette leave size, normal plant height, and normal flower size (Fig. S2a-c). However, the seed size of the *rres1* mutants was slightly larger than that of the wild type (Fig. S2d and e), while the silique length of the *rres1* mutants was shorter than that of the wild type (Fig. S2f and g). To analyze the phenotype of these two *rres1* mutant alleles under salt stress, five-day-old seedlings were transferred from MS medium to 100 mM NaCl medium. Similar to the *19–1*, these two mutant alleles exhibited reduced primary root growth under salt stress (Fig. 1c, d), corroborating that *RRES1* gene is required for salt tolerance in Arabidopsis. Moreover, transformation of *RRES1pro:RRES1 genomic-GFP* fully rescued the salt-hypersensitivity of the *rres1–1* and *rres1–2* mutants (Fig. S3).

We also tested the phenotype of the *rres1* mutants in response to other salts, including 80 mM NaNO_3_, 100 mM KCl, and 80 mM KNO_3_. Both the *rres1–1* and *rres1–2* mutants were hypersensitive to NaNO_3_ (Fig. 1e and f), which was similar to the phenotype of the seedlings grown on NaCl medium. On KCl and KNO_3_ media, the root elongation of the *rres1* mutants was also inhibited, and the growth arrest was much more severe than that under NaCl medium (Fig. 1e and f). Collectively, these results suggested that RRES1 is required for the response to multiple salts.

We then investigated the phenotype of the *rres1* mutant under other abiotic stresses. After being transferred from MS medium to mannitol medium, both the *rres1–1* and *rres1–2* mutants showed an obvious root growth arrest, although the inhibition degree was less severe than that under salt stress (Fig. 1e and f). The hypersensitivity of the *rres1* mutants under osmotic stress could be restored in the complementation lines (Fig. 1e and f), suggesting that RRES1 is also required for osmotic stress tolerance. To test the drought tolerance of the *rres1* mutants, we first performed water loss assay using detached plants. The result showed that the *rres1–1* and *rres1–2* mutants displayed only slightly increased water loss rate compared with the wild type, implying that *RRES1* is not important for drought stress tolerance (Fig. S4a). Consistently, drought tolerance assay performed in soil showed that no significant difference was observed between the wild type and *rres1* mutants after water deprivation (Fig. S4b).

### The *rres1* mutation results in reduced hypocotyl elongation under salt stress

Plants that are hypersensitive to salt stress usually exhibit reduced seed germination under high salinity, so we observed the seed germination and cotyledon greening of the *rres1* mutants after they were sown on NaCl medium. The *rres1* mutants were indistinguishable from the wild type in terms of both seed germination and cotyledon greening under high salinity. Similarly, the *rres1* mutants exhibited normal seed germination when grown on the media supplemented with mannitol or ABA. However, it was noted that both the *rres1–1* and *rres1–2* mutants exhibited decreased cotyledon greening rate when grown on ABA medium (Fig. S5), indicating that *rres1* mutant is partially sensitive to ABA.

**Fig. 2:**
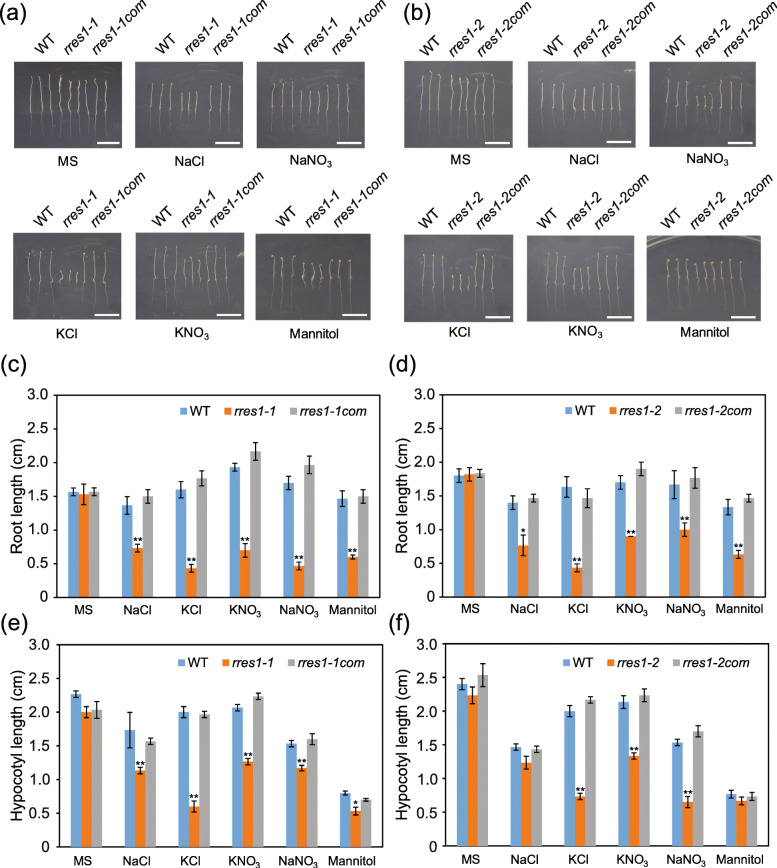
*rres1* mutants exhibit reduced hypocotyl elongation under salt stress. **a-b** Phenotypes of the wild type, *rres1–1*, *rres1–2*, and complementation lines after being transferred to each medium and placed under dark conditions for 7 days. Scale bar = 1 cm. **c-d** Quantification of the hypocotyl length of each genotype shown in (**a**) and (**b**). Values indicate means ± SD (*n* = 6). Asterisks indicate statistically significant differences (*p < 0.05, **p < 0.01 by Student’s *t* test). **e-f** Quantification of the root length of each genotype shown in (**a**) and (**b**). Values indicate means ± SD (*n* = 6). Asterisks indicate statistically significant differences (*p < 0.05, **p < 0.01 by Student’s *t* test)

To determine whether the *rres1* mutation affects the growth of aerial tissues, we investigated hypocotyl elongation under salt stress. To this end, seeds were sown on NaCl medium and placed under dark conditions. After growth for 7 days, hypocotyl length was measured. Compared with the wild type, the *rres1* mutants showed reduced hypocotyl elongation after NaCl treatment, and the phenotype was recovered in the complementation lines (Fig. 2a-d). Similar to the seedlings grown under normal light conditions, the *rres1* mutants grown under continuous dark conditions also showed enhanced root growth inhibition under high salinity (Fig. 2e and f). These results suggested that *RRES1* is required for salt tolerance in whole seedlings. We also tested the phenotypes of the *rres1* mutants and complementation lines grown on NaNO_3_, KCl, and KNO_3_ media under dark conditions. The hypocotyl and root elongation of the *rres1* mutants were both inhibited by these salts, while the complementation lines showed similar phenotypes as the wild type (Fig. 2). In addition, the *rres1* mutants also exhibited slightly impaired hypocotyl elongation under osmotic stress (Fig. 2).

### The *rres1* mutation results in short meristem zone and reduced auxin accumulation under salt stress

**Fig. 3:**
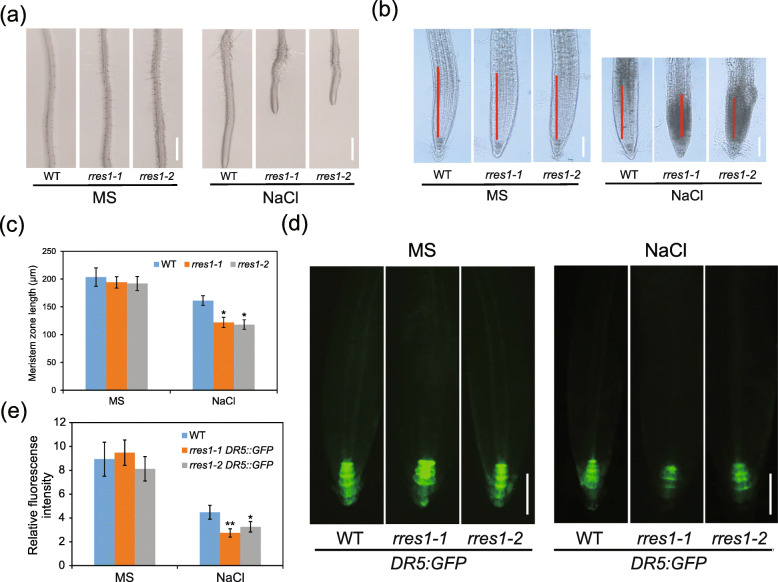
Meristem zone length and auxin accumulation are reduced in *rres1* mutants under salt stress. **a** Root morphology of the wild type, *rres1–1*, and *rres1–2* mutants after being transferred to MS media supplemented with and without 100 mM NaCl was detected by microscopy. Scale bar = 2 mm. **b** Root meristem zone of the wild type, *rres1–1*, and *rres1–2* mutants after being transferred to MS media supplemented with and without 100 mM NaCl. Red lines indicate meristem zones. Scale bar = 60 μm. **c** Quantification of the meristem length of each genotype after being transferred to MS media supplemented with or without 100 mM NaCl. Values indicate means ± SD (*n* = 5). Asterisks indicate statistically significant differences (*p < 0.05 by Student’s *t* test). **d** Detection of the expression of *DR5:GFP* in the wild type and *rres1* mutants with or without salt stress treatment. Fluorescent signals were detected using a Carl Zeiss HBO100 microscope. Scale bar = 70 μm. **e** Quantification of the relative fluorescence intensity of the root tips shown in (**d**). Values indicate means ± SD (*n* = 5). Asterisks indicate statistically significant differences (*p < 0.05, **p < 0.01 by Student’s *t* test)

As *rres1* mutant exhibited reduced root elongation under salt stress, we took a close view of root cells under a microscope. Under normal conditions, both the *rres1–1* and *rres1–2* mutants displayed normal root morphology, but developed more and longer root hairs (Fig. 3a). Under salt stress, however, root elongation was severely inhibited in the *rres1* mutants (Fig. 3a). Moreover, we noted that meristem zone length in the *rres1* mutants was slightly reduced under normal conditions, but it was strongly reduced under salt stress (Fig. 3b and c). Microscopic pictures also showed that meristem zones were more thickened in the *rres1* mutants than that in the wild type after being exposed to salt stress (Fig. 3b and c). These results suggested that RRES1 is required for the maintenance of meristem activity under salt stress.

Auxin plays critical roles in plant root growth by regulating cell division, expansion, and differentiation (Malamy & Ryan, 2001). To understand whether root growth arrest and reduced meristem zone length in the *rres1* mutant under salt stress is caused by the disruption of auxin signaling pathway, auxin-responsive *DR5:GFP* marker was explored to monitor the accumulation and distribution of auxin (Friml et al., 2003). We crossed *DR5:GFP* transgenic plant with the *rres1–1* and *rres1–2* mutants and detected auxin accumulation in the wild type and *rres1* mutants. Without salt treatment, green fluorescence intensity in primary root tips was similar between the wild type and *rres1* mutants (Fig. 3d and e). Under salt stress, green fluorescence intensity was reduced in the wild type, which is consistent with a previous report (Liu et al., 2015). In the *rres1* mutants, however, the salt stress-triggered inhibition of auxin accumulation was enhanced compared with the wild type (Fig. 3d and e), suggesting that RRES1 is required for the maintenance of auxin accumulation under salt stress. To understand the molecular mechanism underlying the regulation of auxin accumulation by RRES1, we searched for potential RRES1-interacting proteins based on the protein-protein interactome Database. We found that WAT1 (Walls Are Thin1), which functions as an auxin transporter (Ranocha et al., 2013), is annotated as an interacting protein of RRES1. To verify this interaction, we performed split-LUC assay in *N. benthamiana*. Unfortunately, no interaction was observed between RRES1 and WAT1, but we found that WAT1 could form a homodimer (Fig. S6).

### The *rres1* mutants exhibit an increased ROS accumulation in roots under salt stress

**Fig. 4:**
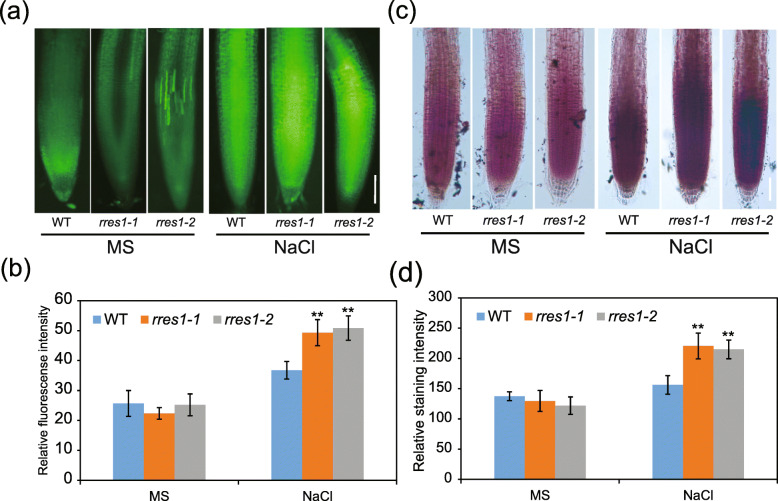
*rres1* mutants exhibit increased ROS accumulation in roots. **a** 2′,7′-dichlorodihy-drofluorescein diacetate (H_2_DCFDA) staining of H_2_O_2_ in the roots of the wild type and *rres1* mutants grown on MS media supplemented with or without NaCl. Scale bar = 30 μm. **b** Relative fluorescence intensity of H_2_DCFDA staining in the roots of the wild type and *rres1* mutants. Values indicate means ± SD (*n* = 5). Asterisks indicate statistically significant differences (**p < 0.01 by Student’s *t* test). **c** DAB staining of H_2_O_2_ in the roots of the wild type and *rres1* mutants with or without NaCl treatment. Scale bar = 20 μm. **d** Relative DAB staining intensity in the roots of the wild type and *rres1* mutants. Values indicate means ± SD (*n* = 5). Asterisks indicate statistically significant differences (**p < 0.01 by Student’s *t* test)

It has been shown that salt stress increases the production of ROS in plant cells, and ROS are involved in the regulation of root elongation (Hazman et al., 2015). Hence, we employed H_2_DCFDA and DAB to detect ROS accumulation in primary root, respectively. ROS levels were similar between the wild type and *rres1* mutants under normal conditions, and salt stress triggered ROS accumulation in both the wild type and *rres1* mutants (Fig. 4a-d). However, both assays supported that the *rres1* mutants accumulated more ROS than the wild type after being exposed to salt stress (Fig. 4a-d), suggesting that RRES1 is a negative regulator of ROS accumulation in response to salt stress.

### Arabinose and xylose contents are decreased in the *rres1* mutant under salt stress

**Fig. 5:**
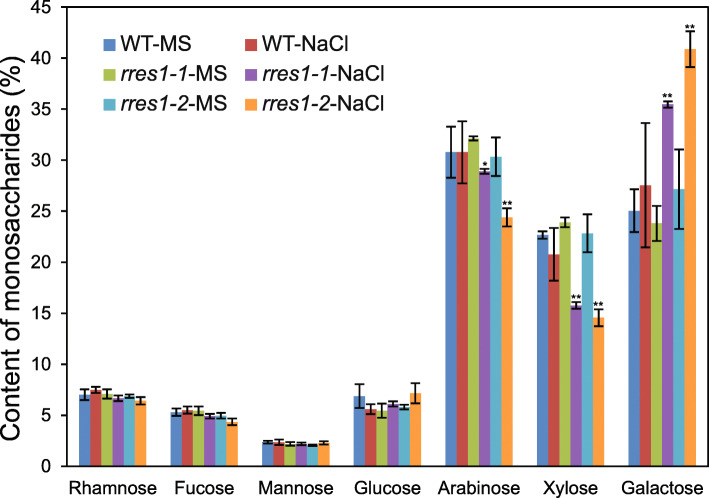
Monosaccharide contents in the roots of *rres1* mutants under salt stress. The seedlings of the wild type, *rres1–1*, and *rres1–2* were treated with or without NaCl, and cell wall components were extracted for the analysis of each monosaccharide content. Values are means ± SD (*n* = 3). Asterisks indicate statistically significant differences (*p < 0.05, **p < 0.01 by Student’s *t* test)

Exposure of plants to salt stress can rapidly induce the compositional and structural changes of cell wall, and the ability to maintain cell wall integrity is critical for plants to tolerate salt stress. As *rres1* mutants exhibited root and hypocotyl elongation defects under salt stress, we speculated that cell wall components are probably affected in the *rres1* mutant. To test this hypothesis, we measured the contents of monosaccharides, including rhamnose, fucose, arabinose, xylose, mannose, glucose, and galactose in the cell wall of the *rres1* mutants. The roots of the wild type and *rres1* mutants before and after salt treatment were collected and cell wall components were extracted to determine the relative amount of each monosaccharide. The contents of rhamnose, fucose, mannose, and glucose were not altered in the *rres1* mutant with or without salt treatment. However, arabinose and xylose contents were significantly reduced in the *rres1* mutants after salt treatment, despite a similar amount of these two monosaccharides was observed between the wild type and *rres1* mutants under normal conditions (Fig. 5). Moreover, compared with the wild type, the relative amount of galactose in the *rres1* mutants was slightly increased under normal conditions and largely increased under salt stress (Fig. 5). Together, these results indicated that RRES1 is involved in controlling the abundance of arabinose, xylose, and galactose, and thus facilitates the maintenance of cell wall integrity under salt stress.

### Boric acid partially restores the short primary roots of the *rres1* mutants under salt stress

**Fig. 6:**
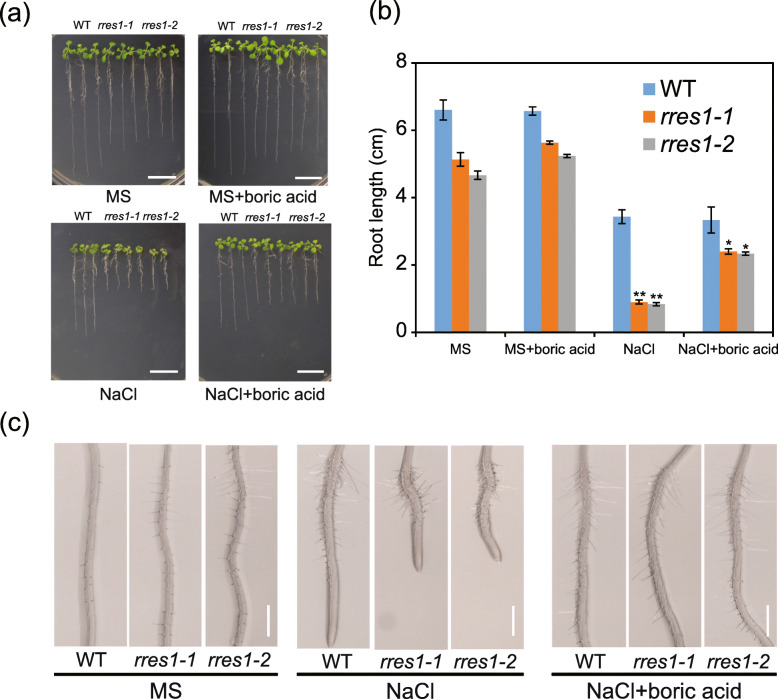
Boric acid partially restores the reduced root elongation of *rres1* mutants under salt stress. **a** Phenotypes of root growth of the wild type, *rres1–1* and *rres1–2* mutants after being transferred to MS and NaCl media supplemented with or without 3 mM boric acid. Photographs were taken after growth for 7 days. Scale bar = 2 cm. **b** Quantification of root length of each genotype shown in (**a**). The length of newly developed roots was measured. Values are means ± SD (*n* = 5). Asterisks indicate statistically significant differences (*p < 0.05, **p < 0.01 by Student’s *t* test). **c** Root morphology of the wild type, *rres1–1* and *rres1–2* mutants after being transferred to NaCl media supplemented with or without 3 mM boric acid. Scale bar = 2 mm

Boric acid is essential for the cross-linking of pectic polysaccharide rhamnogalacturonan-II (RG-II), which in turn promotes cell wall strength (Match, 1997). Studies have shown that application of boric acid can rescue the deficient phenotypes of the mutants that are disrupted in cell wall biosynthesis (O'Neill et al., 2001). To support that the reduced root elongation in the *rres1* mutants under salt stress is caused by cell wall defects, the effect of the exogenous boric acid on the root elongation of the *rres1* mutants was analyzed. On MS medium, the root growth of the *rres1* mutants was slightly increased when boric acid was applied. Under salt stress, the exogenous boric acid had no obvious effect on the root growth of the wild type, but could largely restore the root elongation of the *rres1* mutants (Fig. 6). Assessment of different concentrations of boric acid showed that 3 mM is the optimized concentration to promote the root elongation of the *rres1* mutants under salt stress. The boric acid with a concentration more than 5 mM caused a toxic effect on the root growth of both the wild type and *rres1* mutants (Fig. S7). Together, these results corroborated that the salt-hypersensitive phenotype of the *rres1* mutant is largely attributed to cell wall deficiency.

### RRES1 is localized in the mitochondrion and its gene expression is induced under salt stress

**Fig. 7:**
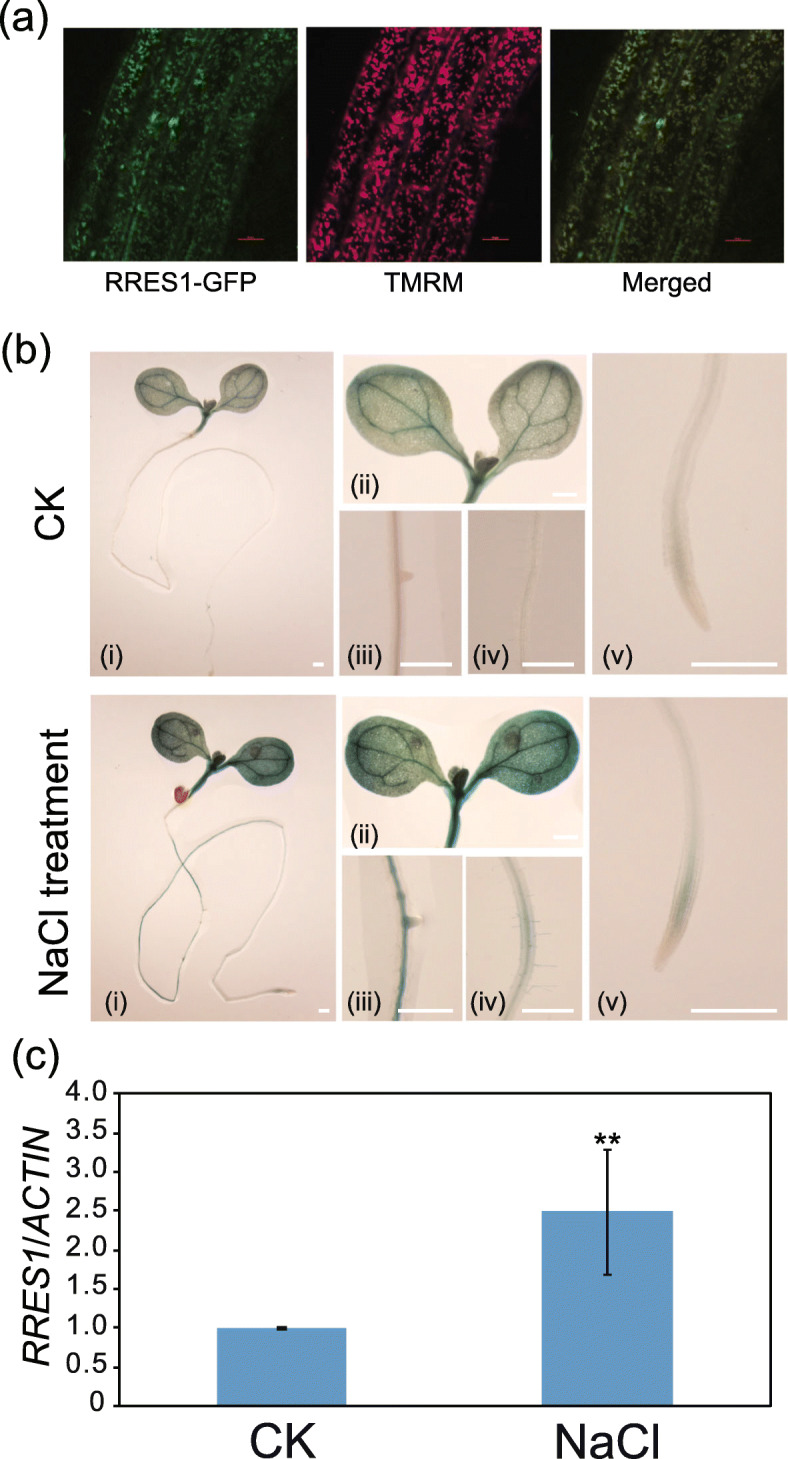
Subcellular localization and tissue-specific expression pattern of *RRES1*. **a** Subcellular localization of the RRES1 fused with GFP tag was examined by confocal microscopy. Tetramethylrhodamine methyl ester (TMRM) was applied as a fluorescence dye of mitochondria. **b** *ProRRES1:GUS* transgenic plants were generated to analyze the expression of *RRES1* in whole seedling (i), cotyledon (ii), mature root (iii), lateral root (iv), and root tip (v) before (upper panel) and after (bottom panel) salt treatment. Scale bar = 2 mm. **c** qRT-PCR analysis of the transcript level of *RRES1* gene before and after NaCl (150 mM) treatment for 6 h. *ACTIN8* was used as an internal control. Values are means ± SD (*n* = 3). Asterisks indicate a statistically significant difference (**p < 0.01 by Student’s *t* test)

Based on the TAIR database, RRES1 is predicted to be localized in mitochondrion. To experimentally determine the subcellular localization of RRES1, we observed fluorescence signal in *RRES1-GFP* transgenic plants by using confocal microscopy. Green fluorescence was found in a large number of discrete puncta (Fig. 7a), which are reminiscent of the structure of mitochondria. We then explored tetramethylrhodamine methyl ester (TMRM), a fluorescent dye that is often used to label mitochondria in living cell. As expected, GFP fluorescence was overlapped with TMRM signal, indicating that RRES1 is localized in the mitochondrion (Fig. 7a).

To determine the tissue-specific expression pattern of the *RRES1* gene, we generated transgenic plants expressing *ProRRES1:GUS*. Under normal conditions, the *GUS* was mainly expressed in leaves and weakly in root tip. After salt treatment for 12 h, however, the GUS staining signal was obviously increased in leaves, lateral root, root hair, and root tip (Fig. 7b), suggesting that *RRES1* promoter is responsive to salt stress. Consistently, qRT-PCR analysis also showed that the expression of *RRES1* gene was significantly up-regulated after salt treatment (Fig. 7c).

## Discussion

After being subjected to salt stress, plants utilize a multitude of mechanisms to maintain root elongation, which could be important for seedling establishment and ensuring adequate supply of water and nutrients for plant growth (Petricka et al., 2012). In addition, maintenance of root growth facilitates plants to escape from high concentration of salts via a process of halotropism response (Galvan-Ampudia & Testerink, 2011). Therefore, tight regulation of root elongation would be beneficial for plant growth and survival under salt stress. In this study, we reveal a novel mitochondrial protein that participates in the regulation of primary root elongation under salt stress via the modulation of cell wall integrity, ROS homeostasis, and auxin accumulation.

Plant cell wall is a polymer matrix that is comprised of cellulose, hemicellulose, pectin, and structural proteins (McNeil et al., 1984). Plant cell wall not only determines plant growth and development, but is also required for response and adaption to environmental stresses. High salinity can cause a significant change of cell wall components and structure, which in turn affects cell division and expansion (Wolf et al., 2012; Draeger et al., 2015). Accumulating evidence indicates that the plants that are not capable to synthesize cell wall components often lose ability to tolerate salt stress. Arabinose and xylose are important constitutes of pectin polymer side chain (Burget & Reiter, 1999; Burget et al., 2003; Harholt et al., 2010), and it has been demonstrated that disruption of UDP-Xyl and UDP-Ara biosynthesis results in severe root growth inhibition under salt stress (Zhao et al., 2019). Here, we found that the *rres1* mutant showed reduced arabinose and xylose contents under salt stress, suggesting that the reduced root elongation of the *rres1* mutant is likely caused by cell wall defects. This result is consistent with the putative function of RRES1 as a nucleotide-diphospho-sugar transferase, although more experimental evidence is required to demonstrate the glycosyltransferase activity of RRES1. Boron plays a structural role in plant cell wall and boron deficiency causes decreased cross-linking of RG-II, which subsequently leads to plant growth inhibition (Hu & Brown, 1994; O'Neill et al., 2004). In this study, we found that exogenous boric acid application promoted the root elongation of the *rres1* mutant under salt stress in a dose-dependent manner, corroborating that RRES1 participates in the regulation of cell wall integrity under salt stress. However, it is well-known that cell wall polymers are synthesized in the Golgi, so how the mitochondrion-localized RRES1 protein engages in cell wall biosynthesis needs to be further addressed.

ROS are important signals that are required for the regulation of plant development and responses to a variety of environmental stresses. Despite the versatile roles, the exact functional mode of ROS in response to abiotic stress is still largely elusive. Salt stress rapidly increases the accumulation of ROS in both shoots and roots and excessive production of ROS causes oxidative damage on plants (Suzuki et al., 2012). In Arabidopsis, ABA-promoted ROS are important retrograde signals in regulating root meristem activity and root growth by controlling auxin signaling pathway (Yang et al., 2014). Furthermore, mutation of catalase-encoding gene *CAT2* results in severe root growth inhibition under H_2_O_2_ treatment, indicating the important role of catalase in maintaining root meristem activity under oxidative stress (Tsukagoshi, 2012). In rice, it was also reported that excessive accumulation of H_2_O_2_ caused by NaCl inhibits root growth (Lin & Kao, 2001). In this study, we found that the *rres1* mutant generated more H_2_O_2_ than that of the wild type after salt treatment, which could be one of the factors that cause root elongation inhibition under salt stress. In plants, ROS are mainly produced in the apoplast, chloroplast, mitochondrion, and peroxisome. Given the mitochondrion localization of RRES1 protein, whether RRES1 mediates the production of ROS in the mitochondrion needs to be further studied.

Auxin plays critical roles in plant root growth by regulating cell division, expansion, and differentiation (Malamy & Ryan, 2001). Salt stress affects auxin accumulation and distribution in root tip, and thus causes reduced root growth (Wang et al., 2009; Liu et al., 2015). *IAR4* encodes a putative mitochondrial pyruvate dehydrogenase E1α-subunit that functions as a regulator of auxin homeostasis. Mutation of *IAR4* does not obviously affect growth under normal conditions, but dramatically decreases root elongation under salt stress, and this phenotype is proposed to be caused by increased ROS production and reduced auxin accumulation (Fu et al., 2019). Consistent with this observation, we found that the salt-triggered decrease of *DR5:GFP* expression was enhanced in the *rres1* mutant, suggesting that *RRES1* is required for the regulation of auxin homeostasis under salt stress. ROS are proposed as important mediators between salt stress and auxin (Fu et al., 2019), so whether RRES1 directly regulates auxin pathway or through ROS-mediated pathway remains to be determined. The well-known function of auxin is to regulate root meristem activity (Liu et al., 2015). In line with the reduced auxin accumulation, the *rres1* mutants exhibited a reduced meristem length under salt stress. Together, these results suggest that the reduced meristem activity in the *rres1* mutant could be responsible for the reduced root elongation under salt stress.

It has been reported that salt-hypersensitive mutants, including *mur4*, *pp2a-c5–1*, *sos5*, and *sos6*, are not only sensitive to Na^+^ but also exhibits increased sensitivity to K^+^ (Shi et al., 2003; Zhu et al., 2010; Hu et al., 2017; Zhao et al., 2019). Similarly, the *rres1* mutant showed reduced root growth under both NaCl and KCl, and within a similar ion concentration, the K^+^ has a more severe effect than the Na^+^ on the root growth inhibition of the *rres1* mutant. In fact, early studies have uncovered that in some plants the impairment caused by K^+^ is more serious than that of Na^+^ (Eshel, 1985; Cramer et al., 1990). In *Chenopodium album*, due to the different patterns of antioxidant response and ion regulation, plant growth is more affected by KCl than that by NaCl (Yao et al., 2010). In some halophytes, plasma membrane ATPase activity is reduced to a greater extent by KCl than NaCl, and this may account for the inhibition of photosynthesis and plant growth caused by KCl (Zhao et al., 1995). Another study elucidated that the severe impairment of plant growth caused by a high concentration of K^+^ is due to the significant decrease of H^+^-pumping activity at the plasma membrane (Liu & Wu, 1999). In future, the mechanism underlying the severe toxicity of high K^+^ on the *rres1* mutant needs to be further investigated.

## Materials and methods

### Plant materials and growth conditions

All Arabidopsis materials used in this study were in Columbia (Col-0) background. Two T-DNA insertion mutants SALK_027606 and SALK_004322 were obtained from the Arabidopsis Stock Center (ABRC). *DR5:GFP* plant was a gift from Dr. Lei Ge (Shandong Agricultural University). Arabidopsis seeds were surface sterilized for 5 min with 1% Sodium hypochlorite, and washed three times with sterile water. The sterilized seeds were sown on half Murashige and Skoog (MS) medium containing 1% sucrose and 0.8% agar and vernalized for 3 d at 4 °C before they were placed in a chamber at 23 °C with long-day light cycle (16 h light/8 h dark). For stress treatment, five-day-old seedlings grown on MS medium were transferred onto plates supplemented with different concentrations of NaCl, KCl, KNO_3_, NaNO_3_, or mannitol. After growth for around 5 days, the seedlings were photographed by a digital camera (PowerShot SX20IS, Canon, Japan). For the quantification of root length, only the newly developed root was measured. Primers used for genotyping are listed in Table S1.

### Generation of transgenic plants

To generate *RRES1* complementation lines, the genomic sequence of *RRES1* containing 2 kb promoter sequence was amplified by high-fidelity DNA polymerase (PrimeSTAR HS DNA Polymerase, Clontech). The PCR product was first introduced into pDONR207 vector (Invitrogen) by BP reaction, and then was cloned into destination vector pMDC107 vector via LR reaction. After verification of the construct by using traditional Sanger sequencing, the construct was transformed to *rres1* mutants via an *Agrobacterium tumefaciens*-mediated floral-dip method.

### Split firefly luciferase (LUC) complementation assay

The full-length coding sequences of *RRES1* and *WAT1* were amplified by PCR using primers listed in Table S1. The PCR products were first cloned into pDONR207 vector using BP clonase II (Invitrogen) and then recombined into nLUC or cLUC vectors via LR reactions. Split-LUC complementation assay was performed in tobacco (*Nicotiana benthamiana*) leaves (Christian et al., 2011). After 48 h, luciferin was sprayed on leaves, and fluorescence was detected using a charge-coupled device (CCD) camera (Prince-ton Instruments, Trenton, NJ, USA). Images and quantification of LUC activity were processed by using WinView software.

### Drought stress assay

Water loss assay was performed by using the aerial part of four-week-old plants grown under normal conditions. For each replicate, three plants were placed on a piece of weighing paper under light conditions, and the weight of the plants was measured every 10 min. The relative change of fresh weight at each time point was calculated to assess water loss rate. Three replicates were performed for each genotype. To perform drought stress assay in soil, seven-day-old seedlings were transferred to soil and grown for 2 weeks under normal conditions, and then plants were subjected to drought treatment by stopping watering for the next 2 weeks.

### Measurement of ROS in plants

Eight-day-old seedlings of the wild type, *rres1–1*, and *rres1–2* were incubated in liquid MS with or without 150 mM NaCl for 12 h before the seedlings were stained. For 3′, 3′-diaminobenzidine (DAB) staining, the seedlings were immersed in 1.0 mg/mL DAB (Sigma-Aldrich) dissolved in 50 mM Tris-HCl (pH 5.0) for 10 h and then washed three times with water. The roots were then photographed using a Carl Zeiss HBO100 microscope. For chloromethyl derivative of 2′,7′-dichlorodihydrofluorescin diacetate (CM-H_2_DCFDA) staining, seedlings were incubated in a buffer containing 10 μM CM-H_2_DCFDA (Sigma-Aldrich) at 37 °C in darkness for 30 min and then washed with distilled H_2_O to remove excess CM-H_2_DCFDA. The roots were then photographed using a Carl Zeiss HBO100 microscope. ROS levels were quantified based on the intensity of fluorescence by using ImageJ software (NIH, http://rsb.info.nih.gov/ij/).

### Measurement of root meristems

Seeds were germinated on half-strength MS medium as described above and five-day-old seedlings were transferred to MS media supplemented with or without 100 mM NaCl. After growth for 5 days, roots were excised and immersed immediately in clean solution (5 g chloral hydrate, 1.5 mL water, and 1 mL glycerol) and then were photographed by using an Olympus BH2 microscope. Root meristem zone was defined according to published approach (Dello et al., 2007), and root meristem length was measured as described previously (Yuan et al., 2013).

### Detection of DR5:GFP fluorescence

Transgenic plants carrying *ProDR5:GFP* were crossed with *rres1–1* and *rres1–2* mutants, and F_2_ seedlings were screened on MS media containing 50 mg/L hygromycin. Meanwhile, hygromycin-resistant seedlings were genotyped for the *rres1* mutations using specific primers (Table S1). The hygromycin-resistant and homozygous F_3_ seeds were sown on MS for 5 days and then were transferred to MS media supplemented with or without 100 mM NaCl, and grown for an additional 8 h. Seedlings were photographed by a Carl Zeiss HBO100 microscope. The intensity of fluorescence was quantified using ImageJ software.

### Quantification of monosaccharides in cell wall

Cell walls were extracted based on the protocol described previously (Mertz et al., 2012). Briefly, seeds were grown on MS medium for 5 days. For salt treatment, half of five-day-old seedlings were transferred onto plates supplemented with 100 mM NaCl. Three biological replicates were performed for each sample. After 15 days, roots were collected and rapidly frozen into liquid nitrogen. The samples were grounded and suspended in 50 mM Tris [HCl] (pH 7.2) mixed with 1% SDS. Suspensions were vortexed and centrifuged at 2500 g for 5 min, and pellets were resuspended in the same buffer and heated at 65 °C for 20 min. After that, the pellets were washed with 50 °C water for three times, with 50% ethanol for three times, and with water for three times. The isolated cell walls were freeze dried for further analysis.

2.0 mg of each cell wall material (totally 18 samples) was carboxyl-reduced with NaBD_4_ and acetylated in 1-Methyl-Imidazole as described previously (Gibeaut & Carpita, 1991). Samples were added with 0.6 ml solution containing 0.5 ml of fresh 20 mg/ml NaBH_4_ in DMSO plus 0.1 ml of 1 M NH_4_OH. Then the samples were incubated at 45 °C in water bath for 90 min with vortex every 30 min. The samples were chilled in a freezer for 15 min and neutralized with 100 μl glacial acetic acid (letting it run slowly down along the side of tubes), and then mixed thoroughly until fizzing stopped. Next 100 μL 1-methylimidazole (4 °C) and 0.75 ml anhydrous acetic anhydribe were added. The tubes were capped tightly and mixed thoroughly before they were incubated at 45 °C in water bath for 30 min. Then add 1.5 ml H_2_O and fill remaining volume with dichloromethane (CH_2_Cl_2_), shake vigorously for 30 s and spin at 2500 rpm for 3 min. Pour off the suspension and refill with H_2_O. The samples were washed with water for five times and then the CH_2_Cl_2_ was evaporated at 45 °C. The last step is to redissolve the samples in 0.5 ml CH_2_Cl_2_ for GC-MS analysis.

### GUS staining assay

*RRES1* promoter (approximately 2 kb) was amplified by PCR and then the PCR product was cloned into binary vector pMDC162 (with a GUS coding region). The binary construct was transformed into wild-type (Col-0) plants via *Agrobacterium*-mediated transformation as described above. Eight-day-old hygromycin-resistant T_2_ transgenic seedlings grown vertically on MS were transferred to the media supplemented with or without 100 mM NaCl and the seedlings were grown for an additional 8 h. For GUS staining, seedlings were incubated in X-Gluc solution (0.5 mg/ML X-Gluc, 0.1% sodium phosphate buffer (pH 7.0), 0.5% Triton X-100, 5 mM K_3_ [Fe (CN)_6_], 5 mM K_4_[Fe (CN)_6_], and 0.02% NaN3) at 37 °C overnight in the dark. Then the seedlings were incubated in 70% ethanol for 12 h to remove chlorophyll and photographed using a Leica EZ4 HD.

### Subcellular localization

Based on the TAIR (www.arabidopsis.org) database, *RRES1* is predicted to be localized in mitochondrion. We performed mitochondrion staining of 10-days-old *RRES1-GFP* transgenic seedlings by using TMRM solution. Images were obtained using a Carl Zeiss LSM510 META laser scanning microscope.

## Supplementary Information

**Additional file 1: Table S1.** Primers used in this study.

**Additional file 2: Fig. S1.** Identification of *19–1* mutant that is hypersensitive to salt stress. (**a**) Root growth of seedlings grown on MS media supplemented with or without NaCl (120 mM) for 7 days. (**b**) Quantification of root length in the wild type and mutant under salt stress. The length of newly developed roots was measured. Values indicate means ± SD (*n* = 5). Asterisks indicate statistically significant differences (*p < 0.05, **p < 0.01 by Student’s *t* test). (**c**) Identification of mutations in the *19–1* mutant by bulk segregant analysis.

**Additional file 3: Fig. S2.** Developmental phenotypes of *rres1* mutants. (**a**) Phenotype of plants grown on soil for 4 weeks. Bar = 3 cm. (**b**) Plant height of each plant after growth for 6 weeks. Bar = 2 cm. (**c**) Flower phenotype of each genotype. (**d**) Pictures showing the seeds of each genotype. (**e**) Quantification of the length and width of seeds. Values indicate means ± SD (*n* = 20). Asterisk indicates a statistically significant difference (*p < 0.05 by Student’s *t* test). (**f**) Silique phenotype of each genotype. (**g**) Quantification of the length of the siliques shown in (**f**). Values indicate means ± SD (*n* = 5). Asterisk indicates statistically significant differences (*p < 0.05 by Student’s *t* test).

**Additional file 4: Fig. S3.** Complementation of *rres1* mutants. (**a**) Phenotypes of the wild type, *rres1–1*, *rres1–2*, and complementation lines after being transferred to MS media supplemented with or without 100 mM NaCl for 7 days. (**b**) Quantification of root length of the wild type, *rres1–1*, *rres1–2*, and complementation lines after being transferred to MS media supplemented with or without 100 mM NaCl for 7 days. The length of newly developed roots was measured. Values are means ± SD (*n* = 5). Asterisks indicate statistically significant differences (**p < 0.01 by Student’s *t* test).

**Additional file 5: Fig. S4.** *rres1* mutants were not sensitive to drought stress. (**a**) Water loss assay of the wild type, *rres1–1*, and *rres1–2* mutants. Nine seedlings that grown on MS media for 10 days we detached from soil and weighed every 10 min under normal conditions. (**b**) Drought tolerance assay of the wild type, *rres1–1* and *rres1–2* mutants grown on soil.

**Additional file 6: Fig. S5.** Seed germination and cotyledon greening rates of *rres1* mutants under stress conditions. (**a**) Seeds germination rate of the wild type, *rres1–1*, and *rres1–2* mutants grown on MS or MS media supplemented with NaCl (120 mM), ABA (3 mM), and mannitol (0.5 M). (**b**) Cotyledon greening rate of each genotype grown on MS or MS media supplemented with NaCl (120 mM), ABA (3 mM), and mannitol (0.5 M).

**Additional file 7: Fig. S6.** RRES1 does not interact with auxin transporter WAT1. Split luciferase complementation assay was explored to test the interaction of RRES1 with WAT1 and also the formation of RRES1 and WAT1 homodimers. The constructs expressing the indicated genes were cotransformed into *N. benthamiana* leaves through *Agrobacterium* infiltration. Luciferase activity was determined at 48 h after infiltration. nLUC represents the N-terminal fragment of firefly luciferase; cLUC represents the C-terminal fragment of firefly luciferase.

**Additional file 8: Fig. S7.** Boric acid rescues the reduced root elongation of *rres1* mutants under salt stress in a concentration-dependent manner. (**a**) Root growth phenotype of the wild type, *rres1–1*, and *rres1–2* after being transferred to NaCl media supplemented with different concentrations of boric acid (0.5 mM, 1 mM, 3 mM, 5 mM, and 10 mM) for 7 days. (**b**) Quantification of the root length of the wild type, *rres1–1*, and *rres1–2* shown in (**a**). Values are means ± SD (*n* = 5). Asterisks indicate statistically significant differences (*p < 0.05, **p < 0.01 by Student’s *t* test).

## Data Availability

All data generated or analysed during this study are included in this published article and its supplementary information files.
