# Publisher Correction: A novel mitochondrial protein is required for cell wall integrity, auxin accumulation and root elongation in Arabidopsis under salt stress

**DOI:** 10.1007/s44154-022-00073-y

**Published:** 2022-12-20

**Authors:** Zheping Yu, Yuying Ren, Jianwei Liu, Jian-Kang Zhu, Chunzhao Zhao

**Affiliations:** 1 Institute of Horticulture, Zhejiang Academy of Agricultural Sciences, Hangzhou, 310021 China; 2 Shanghai Center for Plant Stress Biology, CAS Center for Excellence in Molecular Plant Sciences, Chinese Academy of Sciences, Shanghai, 200032 China

**Correction: Stress Biol 2, 13 (2022)**

**https://doi.org/10.1007/s44154-022-00036-3**

Following publication of this article (Yu et al. 2022), it is noticed that the section ‘Materials and methods’ was placed in a wrong place in the main text of this article due to a typesetting error.

The original article (Yu et al. 2022) was updated.
