# Supplementary material for: A novel mitochondrial protein is required for cell wall integrity, auxin accumulation and root elongation in Arabidopsis under salt stress
Source: Stress Biol. 2022 Feb 8;2(1):13. doi: 10.1007/s44154-022-00036-3 (PMC10441957; doi:10.1007/s44154-022-00036-3)
Supplement: Supplementary file 1 — Additional file 1: Table S1. Primers used in this study. [file 44154_2022_36_MOESM1_ESM.pdf]

Supplementary Table 1

Table S1. Primers used in this study.

| Primer name | Sequence | Usage |
|---|---|---|
| *RRES1* RT1-P1 | TTTTTCTTCGGATTCGGCTACG | semi-quantitave RT-PCR |
| *RRES1* RT1-P2 | AAAAGAAACGCCATTTCCAGCA | semi- quantitave RT-PCR |
| *RRES1* RT2-P3 | AAATCTCATGCTTCCTTTGACG | semi- quantitave RT-PCR |
| *RRES1* RT2-P4 | TTCCATGCCTCGAGCATTAAAC | semi- quantitave RT-PCR |
| LB1.3 | ATTTTGCCGATTTCGGAAC | Genotyping |
| *rres1-1* LP | ACATTGCTTTGTTGGATGGAG | Genotyping |
| *rres1-1* RP | TCGAGAAGAAGACAGAGCTGC | Genotyping |
| *rres1-2* LP | CTGGACAAGGCTTGTCTGATC | Genotyping |
| *rres1-2* RP | ACCTTAGGATCCCTTCACGTG | Genotyping |
| *RRES1* genomic-LP | GGGGACAAGTTTGTACAAAAAAGCAGGC TTCTATGTATTCGCCAACTTAGGAA | Plasmid construction Transgenic plants |
| *RRES1* genomic-RP | GGGGACCACTTTGTACAAGAAAGCTGGG TCGAAATCGTATTTAGTTCTTCCA | Plasmid construction Transgenic plants |
| *RRES1* cds-LP | GGGGACAAGTTTGTACAAAAAAGCAGGC TTCACCATGGGTTCCCGGAGAGATTATC | Plasmid construction Split-LUC assay |
| *RRES1* cds-RP | GGGGACCACTTTGTACAAGAAAGCTGGG TCGAAATCGTATTTAGTTCTTCCA | Plasmid construction Split-LUC assay |
| *WAT1* cds-LP | GGGGACAAGTTTGTACAAAAAAGCAGGC TTCACCATGGCGGATAACACCGATAATC | Plasmid construction Split-LUC assay |
| *WAT1* cds-RP | GGGGACCACTTTGTACAAGAAAGCTGGG TCAACATTGTCCGTTGACTGATGG | Plasmid construction Split-LUC assay |
| *RRES1* promoter-LP | GGGGACAAGTTTGTACAAAAAAGCAGGC TTCTATGTATTCGCCAACTTAGGAA | Plasmid construction GUS assay |
| *RRES1* promoter-RP | GGGGACCACTTTGTACAAGAAAGCTGGG TCTAATATCGTATTTTCAACCTTC | Plasmid construction GUS assay |
