# Supplementary figures and images for: A novel mitochondrial protein is required for cell wall integrity, auxin accumulation and root elongation in Arabidopsis under salt stress
Source: Stress Biol. 2022 Feb 8;2(1):13. doi: 10.1007/s44154-022-00036-3 (PMC10441957; doi:10.1007/s44154-022-00036-3)

## Supplementary Figure 1

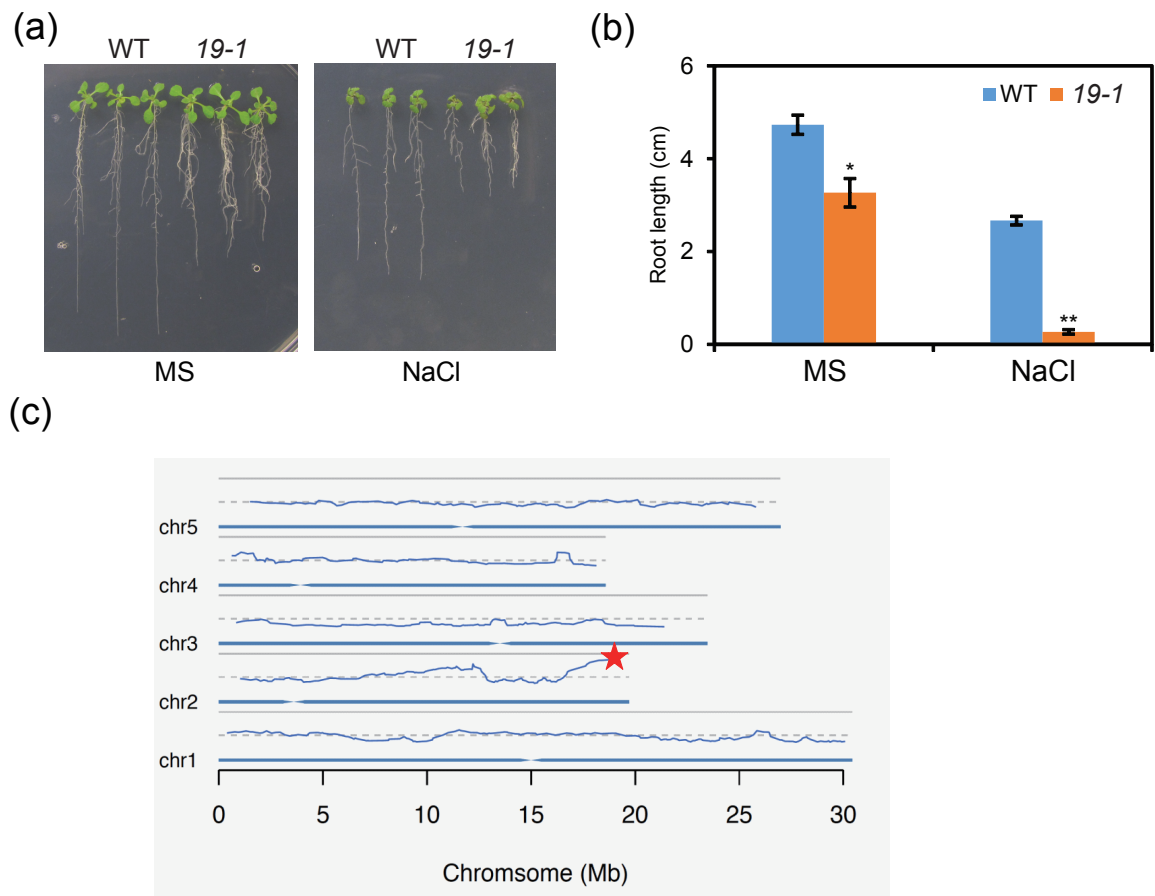

Supplement: Supplementary file 2 — Additional file 2: Fig. S1. Identification of 19–1 mutant that is hypersensitive to salt stress. (a) Root growth of seedlings grown on MS media supplemented with or without NaCl (120 mM) for 7 days. (b) Quantification of root length in the wild type and mutant under salt stress. The length of newly developed roots was measured. Values indicate means ± SD (n = 5). Asterisks indicate statistically significant differences (*p < 0.05, **p < 0.01 by Student’s t test). (c) Identification of mutations in the 19–1 mutant by bulk segregant analysis. [file 44154_2022_36_MOESM2_ESM.pdf]

Supplementary Figure 2

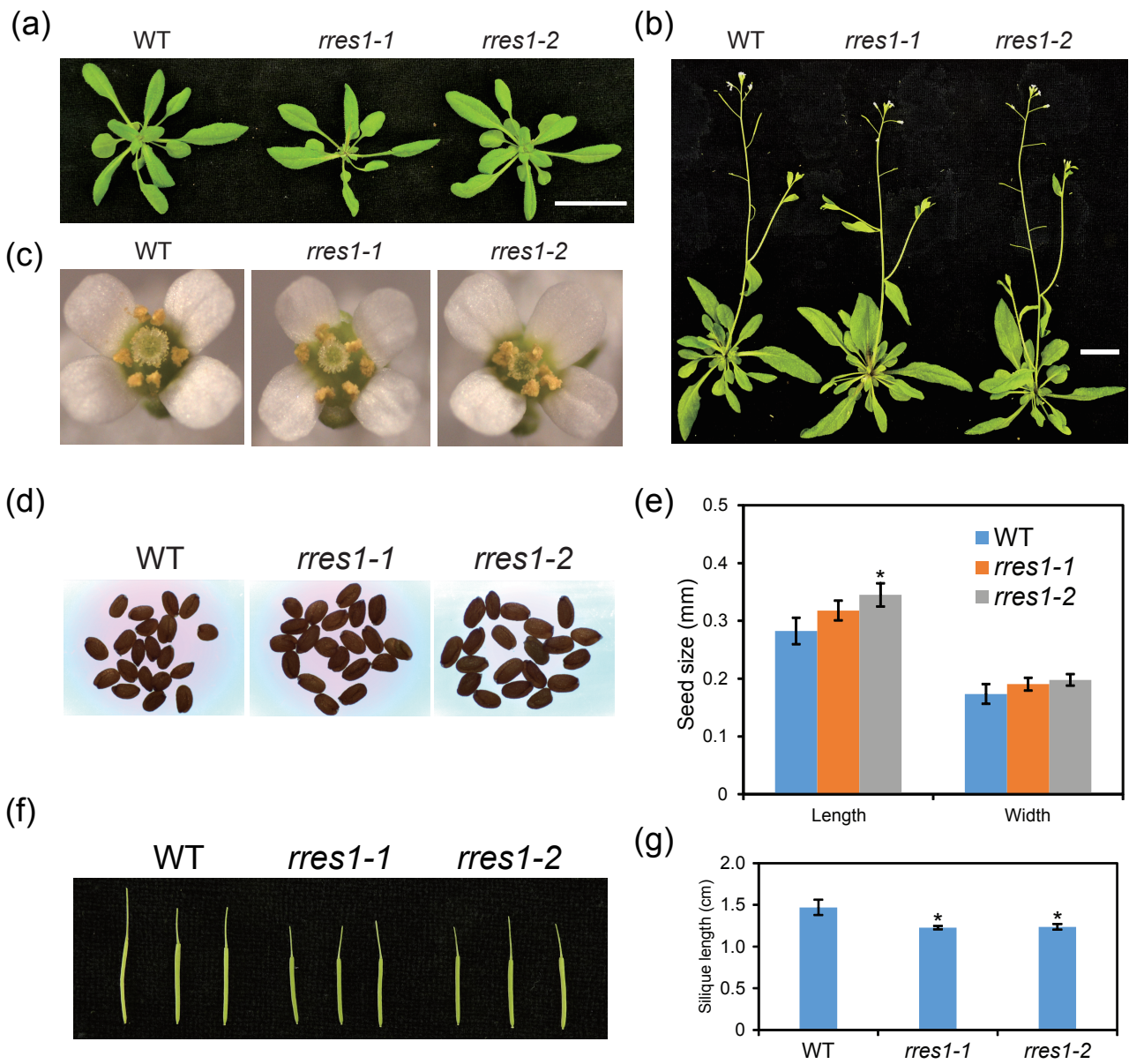

Supplement: Supplementary file 3 — Additional file 3: Fig. S2. Developmental phenotypes of rres1 mutants. (a) Phenotype of plants grown on soil for 4 weeks. Bar = 3 cm. (b) Plant height of each plant after growth for 6 weeks. Bar = 2 cm. (c) Flower phenotype of each genotype. (d) Pictures showing the seeds of each genotype. (e) Quantification of the length and width of seeds. Values indicate means ± SD (n = 20). Asterisk indicates a statistically significant difference (*p < 0.05 by Student’s t test). (f) Silique phenotype of each genotype. (g) Quantification of the length of the siliques shown in (f). Values indicate means ± SD (n = 5). Asterisk indicates statistically significant differences (*p < 0.05 by Student’s t test). [file 44154_2022_36_MOESM3_ESM.pdf]

Supplementary Figure 3

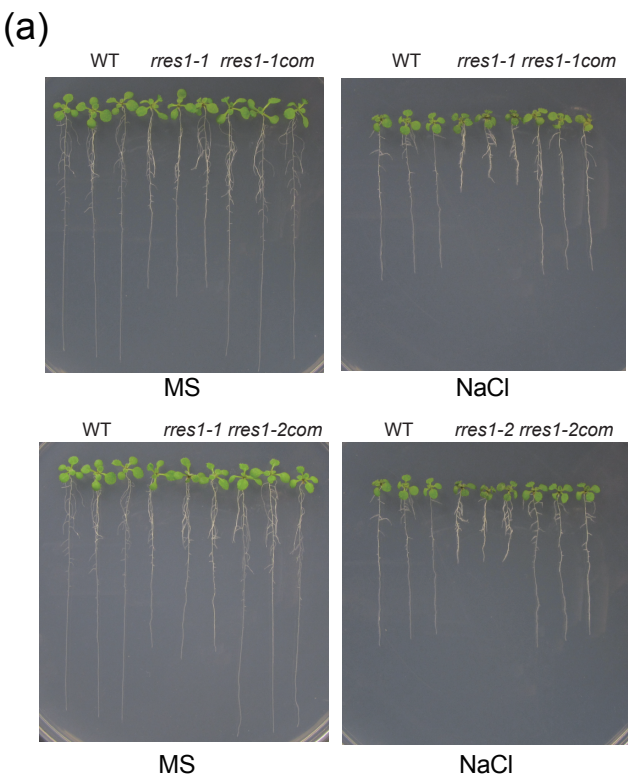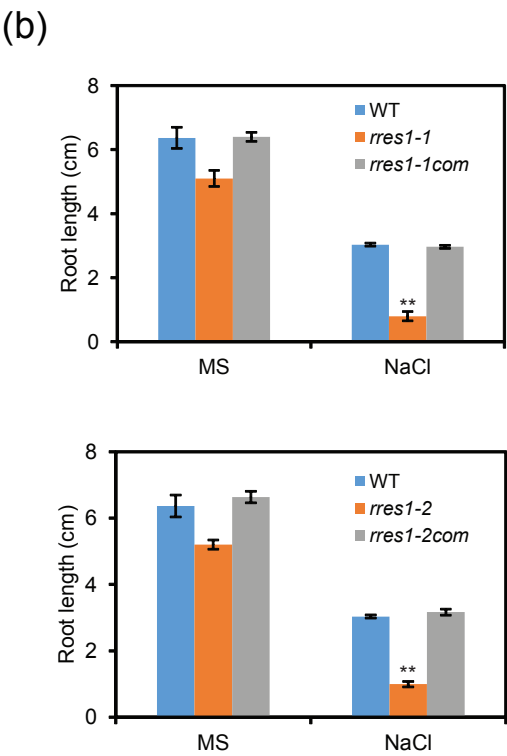

Supplement: Supplementary file 4 — Additional file 4: Fig. S3. Complementation of rres1 mutants. (a) Phenotypes of the wild type, rres1–1, rres1–2, and complementation lines after being transferred to MS media supplemented with or without 100 mM NaCl for 7 days. (b) Quantification of root length of the wild type, rres1–1, rres1–2, and complementation lines after being transferred to MS media supplemented with or without 100 mM NaCl for 7 days. The length of newly developed roots was measured. Values are means ± SD (n = 5). Asterisks indicate statistically significant differences (**p < 0.01 by Student’s t test). [file 44154_2022_36_MOESM4_ESM.pdf]

Supplementary Figure 4

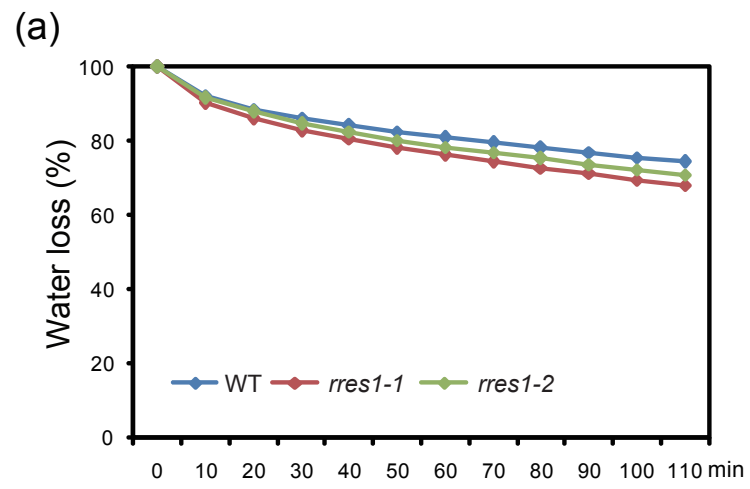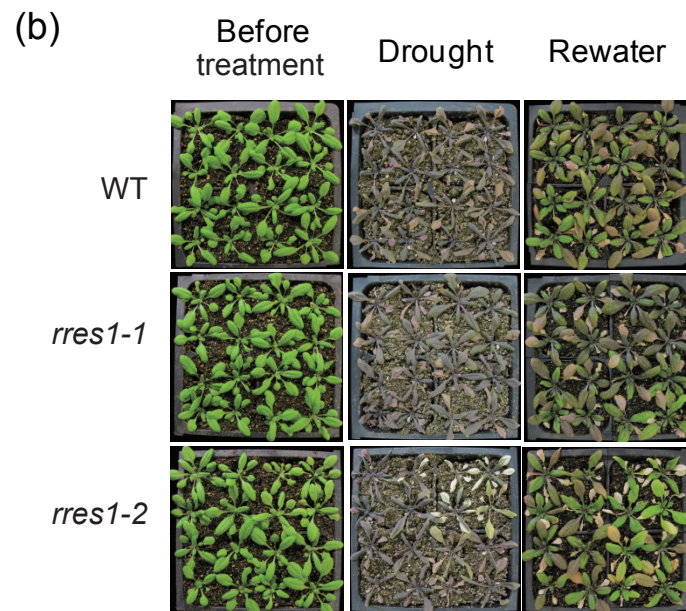

Supplement: Supplementary file 5 — Additional file 5: Fig. S4. rres1 mutants were not sensitive to drought stress. (a) Water loss assay of the wild type, rres1–1, and rres1–2 mutants. Nine seedlings that grown on MS media for 10 days we detached from soil and weighed every 10 min under normal conditions. (b) Drought tolerance assay of the wild type, rres1–1 and rres1–2 mutants grown on soil. [file 44154_2022_36_MOESM5_ESM.pdf]

## Supplementary Figure 5

(a)

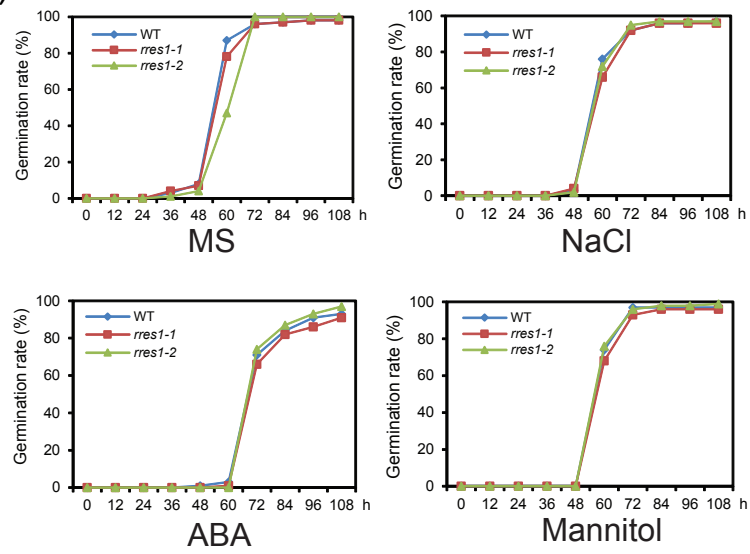

(b)

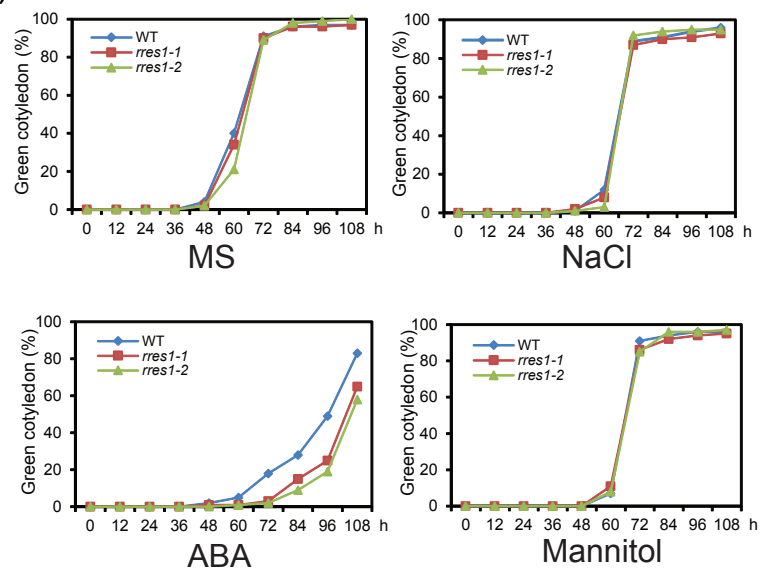

Supplement: Supplementary file 6 — Additional file 6: Fig. S5. Seed germination and cotyledon greening rates of rres1 mutants under stress conditions. (a) Seeds germination rate of the wild type, rres1–1, and rres1–2 mutants grown on MS or MS media supplemented with NaCl (120 mM), ABA (3 mM), and mannitol (0.5 M). (b) Cotyledon greening rate of each genotype grown on MS or MS media supplemented with NaCl (120 mM), ABA (3 mM), and mannitol (0.5 M). [file 44154_2022_36_MOESM6_ESM.pdf]

## Supplementary Figure 7

(a)

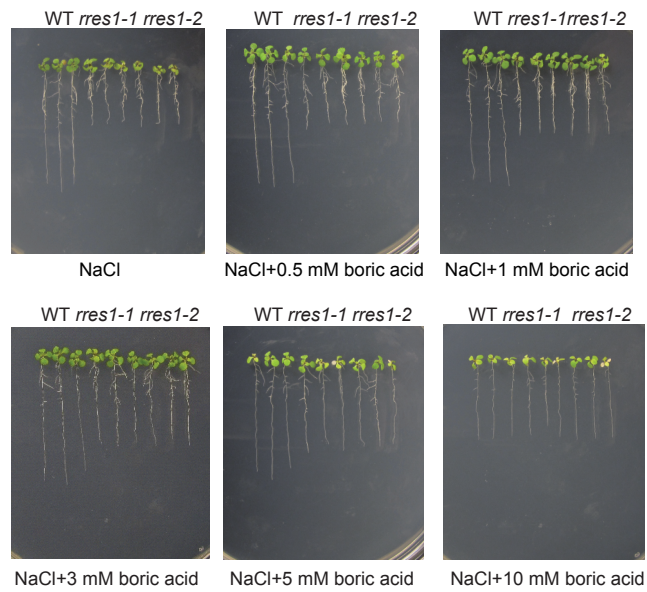

(b)

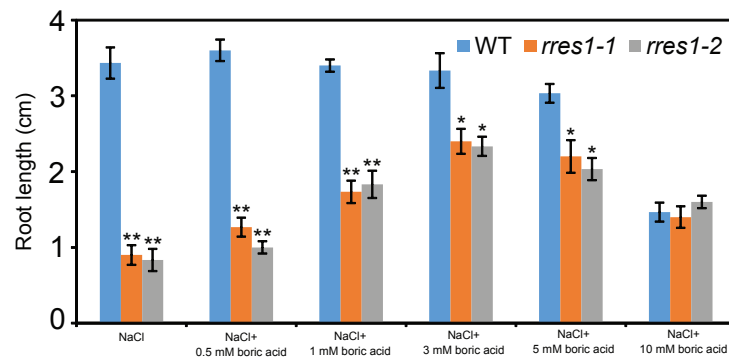

Supplement: Supplementary file 8 — Additional file 8: Fig. S7. Boric acid rescues the reduced root elongation of rres1 mutants under salt stress in a concentration-dependent manner. (a) Root growth phenotype of the wild type, rres1–1, and rres1–2 after being transferred to NaCl media supplemented with different concentrations of boric acid (0.5 mM, 1 mM, 3 mM, 5 mM, and 10 mM) for 7 days. (b) Quantification of the root length of the wild type, rres1–1, and rres1–2 shown in (a). Values are means ± SD (n = 5). Asterisks indicate statistically significant differences (*p < 0.05, **p < 0.01 by Student’s t test). [file 44154_2022_36_MOESM8_ESM.pdf]
