# Supplementary material for: A novel mitochondrial protein is required for cell wall integrity, auxin accumulation and root elongation in Arabidopsis under salt stress
Source: Stress Biol. 2022 Feb 8;2(1):13. doi: 10.1007/s44154-022-00036-3 (PMC10441957; doi:10.1007/s44154-022-00036-3)
Supplement: Supplementary file 7 — Additional file 7: Fig. S6. RRES1 does not interact with auxin transporter WAT1. Split luciferase complementation assay was explored to test the interaction of RRES1 with WAT1 and also the formation of RRES1 and WAT1 homodimers. The constructs expressing the indicated genes were cotransformed into N. benthamiana leaves through Agrobacterium infiltration. Luciferase activity was determined at 48 h after infiltration. nLUC represents the N-terminal fragment of firefly luciferase; cLUC represents the C-terminal fragment of firefly luciferase. [file 44154_2022_36_MOESM7_ESM.pdf]

Supplementary Figure 6

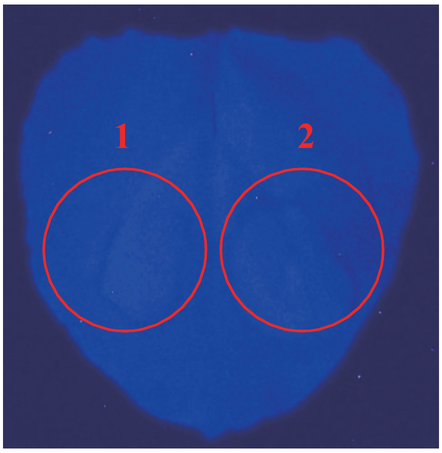

1. nLUC-WAT1+cLUC-RRES1
2. nLUC-WAT1+cLUC-RRES1

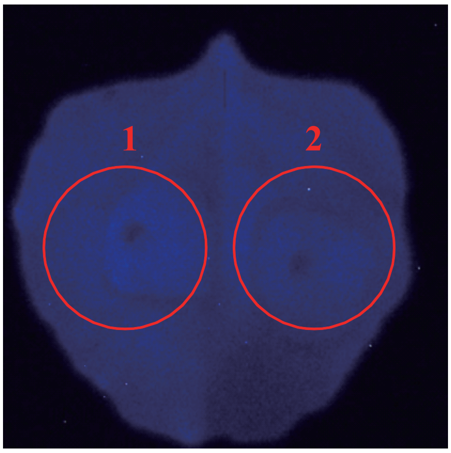

1. nLUC-RRES1+cLUC-RRES1
2. nLUC-RRES1+cLUC-RRES1

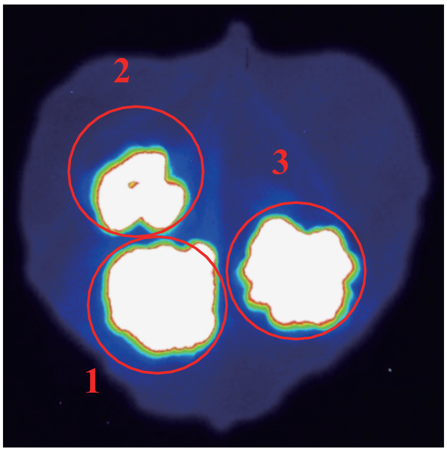

1. nLUC-WAT1+cLUC-WAT1
2. nLUC-WAT1+cLUC-WAT1
3. nLUC-WAT1+cLUC-WAT1
